# PD-L1 Expression in Prostate Cancer: Anatomopathological Features, Methodological Pitfalls, and Therapeutic Potential

**DOI:** 10.3390/ijms27041797

**Published:** 2026-02-13

**Authors:** Ludovica Pepe, Cristina Pizzimenti, Pietro Tralongo, Valeria Zuccalà, Antonio Ieni, Pietro Pepe, Gabriele Ricciardi, Vincenzo Cianci, Cristina Mondello, Maurizio Martini, Guido Fadda, Vincenzo Fiorentino

**Affiliations:** 1 Anatomic Pathology Unit, Department of Human Pathology in Adult and Developmental Age “Gaetano Barresi”, University of Messina, 98125 Messina, Italy; ludopepe97@gmail.com (L.P.); valeria.zuccala@unime.it (V.Z.); antonio.ieni@unime.it (A.I.); maurizio.martini@unime.it (M.M.); guido.fadda@unime.it (G.F.); 2 PhD Program in Translational Molecular Medicine and Surgery, Department of Biomedical Sciences, Dental Sciences and Morpho-functional Imaging, University of Messina, 98125 Messina, Italy; pietrotralongo@gmail.com (P.T.); gricciardi1998@gmail.com (G.R.); 3 Anatomic Pathology Unit, Papardo Hospital, 98158 Messina, Italy; cristinapizzimenti86@gmail.com; 4 Urology Unit, Cannizzaro Hospital, 95126 Catania, Italy; piepepe@hotmail.com; 5 Istituto Clinico Polispecialistico C.O.T. Cure Ortopediche Traumatologiche s.p.a., 98124 Messina, Italy; 6 Section of Legal Medicine, Department of Biomedical Sciences, Dental Sciences and Morpho-Functional Imaging, University of Messina, 98125 Messina, Italy; enzocianci.1997@gmail.com (V.C.); cristina.mondello@unime.it (C.M.)

**Keywords:** PD-L1, prostate cancer, immunotherapy, immune checkpoint inhibitors, biomarker, tumor microenvironment, liquid biopsy, mismatch repair, tumor mutational burden

## Abstract

Programmed death-ligand 1 (PD-L1) has become a central biomarker and therapeutic target across multiple solid tumors, yet its clinical meaning in prostate cancer (PCa) remains unsettled. PCa is commonly described as an immunologically ‘cold’ malignancy, characterized by limited baseline cytotoxic T-cell infiltration and a tumor microenvironment (TME) shaped by myeloid-driven suppression and low neoantigen load in many cases. Against this background, PD-L1 expression in PCa is typically low in untreated primary tumors but can increase in aggressive variants, advanced stages, and metastatic castration-resistant disease, where therapy pressure and microenvironmental cues may select for immune-evasive phenotypes. The literature is further complicated by major analytic variability, including differences in antibody clones and platforms, scoring algorithms (tumor proportion score, combined positive score, immune-cell scoring), cut-offs, tissue sites and timing, and pre-analytical variables such as fixation and decalcification. Collectively, available studies suggest that higher PD-L1 expression tends to be associated with adverse clinicopathological features and may enrich for responses to immune checkpoint inhibitors in selected settings, but PD-L1 immunohistochemistry alone is insufficient as a stand-alone predictive tool in unselected patients. This review synthesizes the biological drivers of PD-L1 regulation in PCa, dissects key methodological sources of heterogeneity in PD-L1 assessment, summarizes clinicopathological and therapeutic correlations, and outlines emerging biomarkers and approaches (including mismatch repair deficiency/microsatellite instability, tumor mutational burden, gene-expression signatures, liquid biopsies, and neuro-immune interactions) that may enable more actionable patient stratification.

## 1. Introduction

Prostate cancer (PCa) is the second most frequent malignancy in males, with an estimated 1.4 million diagnoses and 375,000 deaths worldwide in 2020 [1]. It is the third leading cause of cancer-related mortality among men in Europe and the most common type of cancer diagnosed in men [2].

Blood PSA (prostate-specific antigen) levels and digital rectal examination (DRE) are routinely used to screen for PCa [3,4].

In recent years, the diagnostic pathway for PCa has been revolutionized by the implementation of advanced imaging techniques. Multiparametric Magnetic Resonance Imaging (mpMRI) has emerged as a cornerstone in this field, significantly improving the detection of clinically significant tumors while reducing the overdiagnosis of indolent disease [5], and is also crucial for guiding targeted biopsies [6]. The integration of other advanced imaging modalities, such as PSMA PET/CT, further refines the diagnostic accuracy [7].

Risk stratification systems have been developed for PCa [8], and different treatment choices have been proposed depending on the risk group [9,10].

Low-risk PCa typically undergoes active surveillance, while intermediate-risk PCa is more frequently treated with surgery and different radiation modalities [11]. By contrast, high-risk PCa is often treated with a combination of radiotherapy (RT) and androgen deprivation treatment (ADT) [12].

In fact, ADT has been the standard of care for metastatic PCa. Because of the low survival rates with this monotherapy, various novel therapeutic alternatives have emerged in recent years, such as androgen receptor signaling inhibitors (ARSIs), PARP inhibitors, and Lu-PSMA radioligand therapy. Also, other therapeutic options have been investigated, such as histone lysine demethylase inhibitors [13] and immunotherapy [14,15]. The use of a bispecific T-cell engager (BiTE) in this setting is a novel potential treatment that is not yet established as standard of care [14].

Overall, immunity plays a pivotal role in the host’s response against tumors: in fact, the process of oncogenesis often leads to genetic instability, which culminates in the development of tumor-specific neoantigens that enable the host’s immune response to target malignant cells [16,17].

In such a context, the programmed death-1 (PD-1)/programmed death ligand-1 (PD-L1) axis is highly exploited by tumor cells to evade the host’s immune response. In fact, the binding of PD-L1 to PD-1, a receptor expressed on activated T-cells, activates inhibitory signals, thereby suppressing the T-cell response and enabling tumor cells to evade immune surveillance. The PD-1/PD-L1 axis has therefore become a major target for immune checkpoint inhibitors (ICIs), drugs that restore the host’s immune response against cancer [18].

Globally, the expression of PD-L1 in PCa is a multifaceted phenomenon involving tumor cells, diverse immune cell types and the tumor microenvironment. Understanding the complex patterns of PD-L1 expression in PCa is critical for determining its involvement in tumor biology and its potential role as a therapeutic target [19].

## 2. Tumor Cell PD-L1 Expression

PD-L1 expression has been identified on a variety of immune cell types in the tumor microenvironment, including tumor-infiltrating lymphocyte (TILs), macrophages, and dendritic cells [20,21,22]. The presence of PD-L1-positive immune cells may have a variety of implications, depending on the specific cell type and its spatial distribution within the tumor.

TILs, which are mainly T cells and B cells, are key players in the anti-tumor immune response. PD-L1 expression on TILs has been associated with both positive and negative prognostic outcomes. In some studies, high PD-L1 expression on TILs has been associated with improved survival, suggesting an active immune response against the tumor [23,24]. Conversely, macrophages, which are diverse immune cells capable of both pro- and anti-tumor actions, can also express PD-L1, potentially promoting tumor progression and metastasis. The presence of PD-L1-positive macrophages within the tumor microenvironment (TME) has been associated with poor prognosis in several studies, suggesting a role in tumor development and metastasis [25].

Dendritic cells are professional antigen-presenting cells that play an important role in initiating and regulating the immune response. PD-L1 expression on dendritic cells has been associated with immune suppression and tumor evasion [26].

Immunohistochemistry (IHC) is widely used to assess PD-L1 expression on tumor cells; however, the wide range of antibodies and scoring methods employed across investigations highlights substantial methodological variability in reported PD-L1 positivity in PCa [27,28].

Several factors have been proposed to influence tumor cell PD-L1 expression, including genetic changes, epigenetic modifications, and interactions with the TME. Amplification of the 9p24.1 chromosomal region, which contains the PD-L1 gene, has been associated with increased PD-L1 expression [29]. Furthermore, mutations in key signaling pathways, such as the PI3K/AKT and MAPK pathways, can cause PD-L1 upregulation [30]. Epigenetic modifications, such as DNA methylation and histone acetylation, also impact the regulation of PD-L1 expression [31].

The TME, which consists of stromal cells, immune cells, and soluble factors, can profoundly influence PD-L1 expression on tumor cells. Inflammatory cytokines, such as interferon-gamma (IFN-γ), secreted by immune cells, can induce PD-L1 expression on tumor cells, promoting immune evasion [32]. Hypoxia in the tumor microenvironment may activate hypoxia-inducible factor 1α (HIF-1α), leading to upregulation of PD-L1 expression [33].

Therefore, the expression of PD-L1 on PCa cells is not a static feature but is dynamically regulated by a complex interplay of extrinsic signals from the TME and intrinsic oncogenic pathways. The best-known inducer of its expression is IFN-γ. This cytokine, primarily secreted by activated T cells (especially CD8+ cytotoxic T lymphocytes) and NK cells that have infiltrated the tumor, acts as a signal of an active anti-tumor immune response. This phenomenon is known as “adaptive immune resistance”. When IFN-γ binds to its receptor (IFNGR) on tumor cells, it triggers the phosphorylation and activation of Janus kinases (JAK1/2). These kinases, in turn, phosphorylate and activate the Signal Transducer and Activator of Transcription 1 (STAT1). Activated STAT1 homodimerizes, translocates to the nucleus, and binds to the gamma-activated sequence (GAS) elements in the promoter region of the CD274 gene (the gene encoding PD-L1), directly driving its transcription. This feedback loop allows cancer cells to protect themselves precisely when they are under immune attack, effectively dampening the very response that threatens them [34]. However, several oncogenic pathways frequently dysregulated in PCa have been directly linked to PD-L1 upregulation, independent of IFN-γ. The loss of the tumor suppressor gene PTEN, one of the most common genetic alterations in PCa, leads to the constitutive activation of the PI3K/AKT/mTOR signaling pathway. This activation can increase PD-L1 expression through several mechanisms, including the stabilization of PD-L1 mRNA and increased translation, which is often mediated by mTOR and its downstream effectors. Additionally, while less common than in other cancers, activating mutations in the RAS/MAPK pathway can also promote PD-L1 expression. Activated MEK and ERK kinases can increase the stability and nuclear translocation of transcription factors such as c-Jun and c-Fos (forming the AP-1 complex), which can bind to the CD274 promoter and enhance its expression.

The TME, which consists of stromal cells, immune cells, and soluble factors, can profoundly influence PD-L1 expression on tumor cells and is often characterized by regions of low oxygen, or hypoxia. Hypoxia induces the stabilization of the transcription factor Hypoxia-Inducible Factor 1-alpha (HIF-1α). HIF-1α can directly bind to hypoxia-response elements (HREs) within the CD274 promoter to drive PD-L1 transcription. This allows cancer cells in poorly vascularized regions, which are under metabolic stress, to preemptively shield themselves from immune surveillance. This mechanism is particularly relevant in bulky or poorly perfused tumors.

## 3. Epigenetic Regulation

Epigenetic modifications play a crucial role. Histone deacetylases (HDACs) can suppress PD-L1 expression; therefore, treatment with HDAC inhibitors has been shown in preclinical models to de-repress the CD274 gene, increase PD-L1 levels, and potentially sensitize tumors to immunotherapy. Conversely, DNA hypermethylation of the CD274 promoter can lead to gene silencing, representing another layer of regulation that varies among tumor types. The dynamic nature of these epigenetic marks suggests that epigenetic drugs could be used to modulate the TME in preparation for checkpoint inhibition [35].

## 4. Physiological Function

In a healthy state, the PD-1/PD-L1 axis serves as a crucial immune checkpoint, acting as a “brake” to prevent excessive or prolonged immune responses and maintain self-tolerance. This function is complementary to that of Cytotoxic T-Lymphocyte Antigen 4 (CTLA-4); however, while CTLA-4 primarily regulates the initial priming of T cells in lymph nodes, the PD-1/PD-L1 axis functions mainly within peripheral tissues to limit the activity of effector T cells during inflammatory responses.

PD-1 (CD279) is a cell surface receptor expressed primarily on activated T cells, B cells, natural killer (NK) cells, and monocytes. Its ligand, PD-L1 (also known as B7-H1 or CD274), is a transmembrane protein expressed on a wide range of hematopoietic and non-hematopoietic cells, including T cells, macrophages, dendritic cells (DCs), and various epithelial and endothelial cells, particularly under inflammatory conditions.

When PD-L1 on a healthy cell binds to the PD-1 receptor on an activated T cell, it transmits an inhibitory signal into the T cell. This signal, mediated by the recruitment of phosphatases such as SHP-1 and SHP-2, dampens T-cell receptor signaling, thereby inhibiting T-cell proliferation, cytokine secretion (such as IL-2), and cytotoxic activity. This mechanism is essential for preventing autoimmune reactions in which the immune system might otherwise attack the body’s own tissues.

## 5. Mechanism of Immune Evasion

The regulatory system of the PD-1/PD-L1 checkpoint is nefariously exploited by cancer cells as a primary mechanism of immune evasion. By upregulating the expression of PD-L1 on their cell surface, tumors create a “molecular shield” that directly engages with PD-1 on tumor-infiltrating lymphocytes (TILs). This interaction delivers the potent inhibitory signal described above, effectively paralyzing the anti-tumor T-cell response at the tumor site and allowing the cancer cells to escape immune-mediated destruction. The tumor actively adapts to the pressure from the host’s immune system through this immunological escape process; it is not passive.

**Figure 1:**
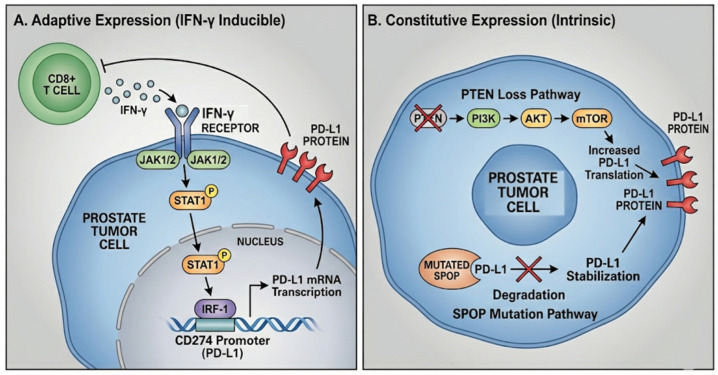
**Regulation mechanisms of PD-L1 expression in PCa.** This diagram illustrates the two primary mechanisms governing PD-L1 expression in prostate tumor cells: adaptive regulation driven by extrinsic immune signals and constitutive regulation driven by intrinsic tumor cell alterations. (**A**) The adaptive expression pathway (IFN-γ inducible) depicts the response to immune attack, where an infiltrating CD8+ T-cell secretes IFN-γ that binds to the IFN-γ receptor on the tumor cell surface. This binding activates the downstream JAK1/2 and STAT1 signaling cascade, causing phosphorylated STAT1 to translocate into the nucleus and induce the expression of the transcription factor IRF-1. IRF-1 subsequently binds to the CD274 promoter, initiating PD-L1 mRNA transcription and leading to the synthesis and surface expression of the PD-L1 protein. (**B**) The constitutive expression panel highlights tumor-intrinsic pathways independent of external inflammation. The functional loss of the tumor suppressor *PTEN* results in the hyperactivation of the PI3K/AKT/mTOR pathway, which leads to increased PD-L1 translation. Alternatively, mutations in the *SPOP* gene produce a protein that fails to mark PD-L1 for degradation, thereby preventing its breakdown and causing PD-L1 stabilization and accumulation on the cell membrane.

The upregulation of PD-L1 on tumor cells is driven by two principal mechanisms (Figure 1). The first is adaptive (inducible) expression, which represents the most common mechanism in immunologically “hot” or inflamed tumors. It is a dynamic reaction to an ongoing immunological attack against the tumor. Activated TILs release a range of cytokines, including IFN-γ, in response to tumor-specific antigens presented by cancer cells. IFN-γ signaling activates the interferon regulatory factor 1 (IRF-1) response element found in the promoter region of the PD-L1 gene (CD274). As a result, IFN-γ exposure causes tumor cells (and other TME cells) to express more PD-L1. A negative feedback loop is thus produced, whereby the tumor expresses PD-L1 to suppress the immune system’s attack. A focal or peripheral pattern of PD-L1 staining, mainly at the interface between tumor cell nests and the surrounding immune infiltration, is frequently used histopathologically to describe this adaptive resistance.

Conversely, the second mechanism involves constitutive (oncogene-driven) expression. Unlike the dynamic, inflammation-driven approach, some tumors have constitutive expression of PD-L1, which results from intrinsic oncogenic signaling pathways that are built into their malignant programming. In PCa, for instance, elevated PD-L1 expression has been connected to loss of the tumor suppressor PTEN, which causes the PI3K/AKT pathway to become hyperactivated. Similarly, robust, diffuse PD-L1 expression is linked to mutations in the SPOP gene, which frequently occur in primary PCa [36]. Histopathologically, this kind of oncogene-driven expression is frequently identified by a diffuse staining pattern throughout the tumor. Crucially, it can also happen without noticeable T-cell infiltration.

The complexity of the axis is further deepened by additional interactions. PD-L1 can engage in “reverse signaling” back into the tumor cell upon binding to PD-1, activating pro-survival and proliferative pathways like PI3K/AKT and MAPK, thus directly contributing to tumor progression independent of its immune-suppressive role. Additionally, PD-L1 can attach to T-cells’ co-stimulatory protein CD80 (B7-1), which inhibits T-cells and adds another layer of immunosuppression to the TME. The difference between constitutive and adaptive PD-L1 expression is not just a theoretical one; it is a basic biological differential with significant therapeutic implications. An adaptive expression pattern suggests a pre-existing, albeit suppressed, anti-tumor immune response that might be readily unleashed by checkpoint blockade. A constitutive pattern, particularly in an immune-excluded or “cold” environment, suggests that checkpoint blockade alone may be insufficient and that therapies targeting the underlying oncogenic driver may be necessary to overcome resistance [36,37,38]. This biological distinction helps explain the heterogeneous clinical activity of ICIs in PCa and provides a rationale for both the biomarker standardization challenges discussed next (Section 6, Section 7 and Section 8) and the TME-priming combination strategies summarized in Section 15.

## 6. The Immunological Landscape of PCa: A “Cold” TME

The TME of PCa is typically regarded as immunologically “cold,” presenting a significant challenge for immunotherapy. This is a complex trait that is characterized by multiple salient qualities. To start, PCa usually has a low tumor mutational burden (TMB), which means that it has fewer somatic mutations than “hot” cancers like lung cancer or melanoma. A lower TMB means that there are fewer mutant proteins that can be converted into neoantigens, which are new peptides that the immune system can identify as alien. It is naturally more difficult to generate a strong, tumor-specific T-cell response when there are fewer neoantigens to target.

In addition, histological examination of most primary prostate tumors reveals a “non-inflamed” phenotype, characterized by a sparse infiltration of immune cells, particularly cytotoxic CD8+ T-cells. The TME is often dominated by immunosuppressive cell populations, such as regulatory T-cells (Tregs) and myeloid-derived suppressor cells (MDSCs), which further dampen any potential anti-tumor response.

In primary, untreated PCa, baseline PD-L1 expression is frequently minimal or absent, consistent with a non-inflamed milieu in which the PD-1/PD-L1 checkpoint is not persistently engaged. Clinically, this means that PD-L1 negativity in an archival diagnostic specimen should not be assumed to reflect the metastatic, treatment-exposed state, where therapy-induced inflammation and clonal evolution can alter PD-L1 expression. This immunological landscape offers a convincing explanation for the generally unsatisfactory outcomes of ICI treatment in large, unselected clinical trials of individuals with PCa.

However, it is crucial to recognize that this “cold” designation is a generalization. A subset of prostate tumors may develop a more inflammatory phenotype, especially those with particular genetic characteristics like mismatch repair deficiency, or those that have undergone therapeutic treatments like radiation or androgen restriction therapy. In these contexts, the PD-1/PD-L1 axis may become biologically significant and therapeutically targetable, underscoring why accurate and standardized assessment is essential for both patient selection and cross-study comparability.

**Figure 2:**
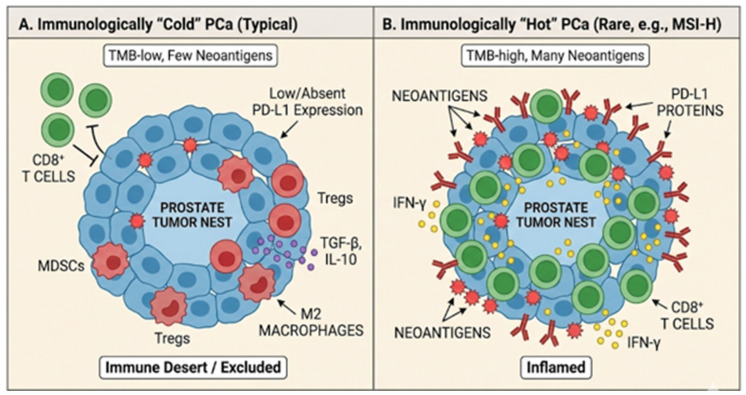
**The immunological landscape of PCa: “cold” vs. “hot” phenotypes.** This figure illustrates the striking TME heterogeneity in PCa. Panel (**A**) represents the predominant phenotype in advanced PCa. It is characterized by a low TMB and sparse neoantigens, failing to attract a significant adaptive immune response. The TME is an “immune desert” or excluded landscape, where effector CD8+ T Cells are trapped in the stroma surrounding the tumor nest. The environment is highly immunosuppressive due to the presence of Tregs, MDSCs, and M2 Macrophages, along with inhibitory cytokines like TGF-β and IL-10. Consequently, PD-L1 expression is typically low or absent. Panel (**B**) represents a rare subset of tumors (e.g., those with Microsatellite Instability-High, MSI-H). A high TMB generates numerous neoantigens, triggering an “inflamed” TME. Active CD8+ T cells successfully infiltrate the tumor nest and secrete IFN-γ. This inflammatory signal drives high adaptive expression of PD-L1 proteins on the surface of both tumor cells and infiltrating immune cells as a counter-regulatory mechanism.

Figure 2 summarizes the main immune microenvironment patterns in PCa (immune-desert/immune-excluded versus inflamed) and their implications for PD-L1 heterogeneity and responsiveness to ICIs.

## 7. Antibody Clones and Staining Platforms

Numerous unique monoclonal antibody clones that were created in conjunction with certain medicinal drugs are employed in research and clinical diagnostics. Every clone has distinct performance traits and identifies a different epitope on the PD-L1 protein, resulting in varying staining patterns and sensitivities. The most prominent clones include 22C3 (Dako/Agilent), an FDA-approved companion diagnostic for the anti-PD-1 drug pembrolizumab that binds to an extracellular epitope [39], and 28-8 (Dako/Agilent), an FDA-approved complementary diagnostic for the anti-PD-1 drug nivolumab, which also targets an extracellular epitope [40]. Regarding the Ventana platform, SP142 (Ventana/Roche) is an FDA-approved companion diagnostic for the anti-PD-L1 drug atezolizumab that targets a cytoplasmic epitope and is known to stain fewer tumor cells but more immune cells compared to other clones [41], while SP263 (Ventana/Roche) is an FDA-approved companion diagnostic for the anti-PD-L1 drug durvalumab, targeting a cytoplasmic epitope identical to that of SP142. Finally, concerning research-use-only clones, a variety of other clones, such as E1L3N, 5H1, and ABM4E54, are widely used in research settings but are not standardized for clinical use [42]. As summarized in Table 1, the major clinical clones used in PCa studies differ in epitope, platform, and scoring readouts, and their interchangeability cannot be assumed.

The choice of antibody clone has a dramatic and demonstrable impact on reported positivity rates in PCa. Maule et al. compared the performance of 22C3, 28-8, and SP142 IHC assays in several tumors, demonstrating that 22C3 is the most sensitive PD-L1 IHC assay for tumor cell expression, followed by 28-8 and, in turn, by SP-142 [43]. Results from studies that have not used the same test cannot be properly compared due to this order-of-magnitude variance based only on the primary antibody employed. The 22C3, 28-8, and SP263 clones are generally concordant for labeling tumor cells, according to extensive harmonization studies in different malignancies, such as the “Blueprint Project” in non-small cell lung cancer (NSCLC). The literature on PCa noticeably lacks these important harmonization attempts [44,45].

From a clinical and trial-design perspective, this analytical variability can shift a case across a binary PD-L1 threshold; consequently, a patient classified as PD-L1-positive in one laboratory may be called negative in another, with direct consequences for eligibility and stratification in biomarker-enriched trials. This problem is further amplified by the heterogeneity in scoring algorithms and cut-offs discussed in Section 8.

## 8. Heterogeneity in Scoring Systems and Cut-Offs

Even if a single antibody clone were universally adopted, the problem of interpretation would remain due to the lack of a standardized scoring system for PCa. Different studies and clinical trials employ a variety of scoring algorithms, each of which measures a distinct aspect of PD-L1 expression and uses different positivity cut-offs. Among these, the Tumor Proportion Score (TPS) is defined as the percentage of viable tumor cells showing at least partial membrane staining of any intensity. It is a measure of tumor-intrinsic PD-L1 expression, and studies in PCa have used a wide range of TPS cut-offs to define positivity, including ≥1%, ≥5%, ≥25%, and ≥50%, each yielding vastly different positivity rates [42,46,47,48,49,50,51,52,53,54,55,56,57]. Alternatively, the Combined Positive Score (CPS) metric is calculated by dividing the total number of PD-L1-staining cells (which includes tumor cells, lymphocytes, and macrophages) by the total number of viable tumor cells, then multiplying by 100. CPS is designed to capture both tumor-intrinsic and adaptive, immune-driven PD-L1 expression [58,59]. Finally, regarding other scores that have been used in other tumors, the Immune Cell (IC) Score quantifies PD-L1 expression on immune cells, typically as the percentage of the tumor area occupied by staining immune cells. This is the primary metric for the SP142 assay in some indications [60]. The variability in clones, platforms, and these scoring approaches across PCa studies is summarized in Table 1.

A significant number of research studies use vague, qualitative, or semi-quantitative scoring systems, such as “low vs. high” based on a median split, or “moderate to high expression,” which are not reproducible and have no clinical validation. The choice between TPS and CPS is particularly consequential. TPS assesses the tumor as an isolated entity, while CPS evaluates the tumor within its immunological context. A tumor could be TPS-negative but CPS-positive if it is surrounded by PD-L1-expressing immune cells. Therefore, these two scores measure fundamentally different biological phenomena, and results derived from them are not interchangeable.

Temporal heterogeneity is a further feature of expression. Over the course of the illness and in response to treatment, PD-L1 status may change. The PD-L1 status of the metastases, which are the final target of therapy, may not be impacted by a primary tumor biopsy performed years before metastatic illness manifests. Research has shown that primary tumors and their associated metastases have different PD-L1 levels [61]. The last problem is inter-observer variability. The same slide can be interpreted differently by pathologists even when the assay and scoring system are consistent. This inconsistency is especially noticeable when scoring immune cells, which can be challenging to differentiate from stromal or tumor cells, and when scoring close to a clinically significant cut-off (e.g., determining if a case is 0.5% or 1.5% positive).

These layers of methodological inconsistency, which include pre-analytics, analytical antibody selection, scoring algorithms, and biological and human variability, combine to produce a perfect mixture of misunderstanding. The “controversy” surrounding PD-L1’s involvement in PCa is clearly not a straightforward biological issue with a clear-cut solution. Instead, the use of non-standard instruments on a complicated biological system is the cause of this metrological disaster. Importantly, these interpretive differences can directly alter whether a patient is categorized as PD-L1-positive around commonly used thresholds, affecting enrollment and stratification in clinical trials and undermining cross-study comparability. The true therapeutic benefit of PD-L1 cannot be demonstrated or disproved unless the field commits to rigorous harmonization and validation efforts, akin to those conducted in lung cancer [62,63,64]. These issues are further compounded by spatial heterogeneity, discussed in Section 9.

## 9. PD-L1 Expression and Spatial Heterogeneity

PD-L1 expression in PCa is not distributed equally throughout the tumor. Several investigations have shown spatial heterogeneity, characterised by differences in PD-L1 expression within distinct tumor areas, as well as in metastases [65,66]. Since biopsy samples might not accurately reflect the tumor’s overall PD-L1 expression status, this heterogeneity could make PD-L1 measurement and interpretation more difficult.

## 10. PD-L1 Expression and Clinicopathological Correlates

Despite the success of PD-1/PD-L1 blockade in several tumors, its role in PCa remains enigmatic and deeply controversial. This review will critically dissect the extensive and often conflicting body of evidence surrounding PD-L1 expression in PCa. It will systematically analyze the relationship between PD-L1 and the canonical pathological markers of tumor aggressiveness, with a primary focus on the Gleason grading system and a comprehensive survey of other key histopathological findings, including pathological stage, perineural invasion, and lymphovascular invasion.

The central thesis is that while a discernible trend associates higher PD-L1 expression with more aggressive PCa phenotypes, this relationship is profoundly confounded by a crisis of methodological heterogeneity in its detection and interpretation. The absence of standardized assays, scoring systems, and positivity thresholds has created a fragmented and often contradictory literature, making the current utility of PD-L1 as a standalone prognostic or predictive biomarker in PCa highly questionable. This report will argue that a more nuanced, multi-parametric, and dynamic understanding of PD-L1 biology is required for its future clinical application. By synthesizing data from primary research articles, comprehensive systematic reviews, and quantitative meta-analyses, this report will aim to provide a definitive, expert-level overview of the state of the science, clarifying points of consensus, deconstructing areas of controversy, and outlining a path forward for research and clinical development.

Several studies have examined the relationship between PD-L1 expression and numerous clinicopathological characteristics in PCa. Tumor cell PD-L1 expression has been consistently connected to aggressive characteristics such as increased Gleason Score (GS), advanced tumor stage, lymph node metastasis, and biochemical recurrence [48,67,68].

The correlation between PD-L1 expression on immune cells and clinicopathological characteristics is more complicated and dependent on the situation. High PD-L1 expression on TILs has been linked to both positive and negative outcomes, depending on the study and TIL subgroup examined [69,70]. PD-L1-positive macrophages have been associated with a poor prognosis, although the prognostic value of PD-L1 expression on dendritic cells is unclear [71,72].

## 11. PD-L1 as a Predictive Biomarker for Immunotherapy

The development of ICIs that target the PD-1/PD-L1 axis has created new opportunities for cancer immunotherapy. In PCa, ICIs have shown encouraging effects in a subgroup of patients with metastatic castration-resistant PCa (mCRPC), resulting in long-term responses and better survival [40,58]. However, response rates to ICIs remain modest, emphasising the need for solid predictive biomarkers for identifying individuals most likely to benefit from this treatment.

One potential predictive biomarker for the ICI response in PCa is PD-L1 expression. Several studies have demonstrated that elevated PD-L1 expression, whether on tumor or immune cells, is related to better response rates and survival outcomes after ICI therapy [73,74]. The association is physiologically reasonable, since PD-L1 expression on tumor cells might directly drive immune evasion, while PD-L1 expression on immune cells may indicate an active immune response to the tumor.

Shen H. et al. investigated the clinicopathological and predictive impact of PD-L1 expression in PCa. The research included 3133 individuals from 15 trials. The findings revealed that increased PD-L1 expression was substantially linked with aggressive clinicopathological characteristics such as higher GS, advanced T stage, lymph node metastasis, and distant metastasis. Furthermore, elevated PD-L1 expression was an independent predictor of poor overall survival, cancer-specific survival, and disease-free survival. These data indicate that PD-L1 expression might be a good predictive biomarker in PCa [75].

Bishop JL et al. investigated the overexpression of immunotherapy targets in CRPC patients, especially PD-L1/2, PD-1, and CTLA-4. The results revealed that patients who improved on enzalutamide had more PD-L1/2+ dendritic cells and a higher frequency of PD-1+ T cells, and this form of resistance was related to high expression of anti-PD-1 treatment targets in circulating immune cells. This discovery implies that PD-L1 may play a role in the development of resistance to enzalutamide, indicating that ICIs might be a suitable target for immunotherapy in this situation [76].

Another study of 23 individuals with castration-resistant PCa discovered that pembrolizumab offered a long-lasting objective response in a subgroup of patients with advanced solid tumors. According to the trial, patients were given pembrolizumab 10 mg/kg every 2 weeks for up to 24 months until disease progression or severe toxicity. The median patient age was 65 years, and 73.9% had received at least two previous treatments for metastatic illness. The study concluded that pembrolizumab produced a long-lasting objective response and had a good side effect profile [77].

Antonarakis ES et al. conducted a study on pembrolizumab in mCRPC. The study involved three cohorts of patients with mCRPC treated with docetaxel and targeted endocrine therapies. Patients were divided into three groups: those with PD-L1-positive and PD-L1-negative disease, and those with bone-predominant disease, regardless of PD-L1 expression. The objective response rate according to RECIST v1.1 was the main outcome. Disease control rate, response time, overall survival, and safety were secondary endpoints. In a subgroup of patients with mCRPC who had previously received docetaxel and targeted endocrine therapy, the trial discovered that pembrolizumab monotherapy had antitumor efficacy with a tolerable safety profile [58].

Noori M. et al. reviewed and evaluated the efficacy and safety of ICIs for PCa; 15 randomized controlled trials with 8922 people were included in the study. The results showed that ICIs, including PD-1/PD-L1 inhibitors and CTLA-4 inhibitors, demonstrated significant antitumor activity in terms of overall survival, progression-free survival, and objective response rate compared to standard therapies. The safety profile of ICIs was generally manageable, with the most common adverse events being fatigue, rash, and diarrhea. This study suggests that ICIs hold promise as a treatment option for PCa [78].

A recent study by Fiorentino V. et al. investigated the relationship between PD-L1 expression and PCa aggressiveness, measured by Gleason Grade Groups (GGs). A retrospective analysis of 120 prostate biopsies from 52 PCa patients revealed a non-linear correlation between CPS positivity and increasing GG, with the highest proportion in GG2 and GG5. GG5 exhibited the highest PD-L1 expression. Heterogeneity was indicated by the variation in PD-L1 expression among patients. Overall, statistical analysis supported a positive link despite the non-linear correlation, with greater GGs typically indicating higher CPS values. A significant association was also observed between cribriform morphology and CPS ≥ 1, and patients with CPS ≥ 1 had a shorter biochemical recurrence (BCR)-free survival. The study highlights a positive correlation between PD-L1 expression and GG in PCa, suggesting that higher PD-L1 expression is linked to more aggressive disease. This discovery may have consequences for immunotherapy use and treatment choices, especially for patients with greater GGs [79].

However, some investigations have shown no apparent connection between PD-L1 expression and ICI responsiveness [74]. This might be due to a variety of factors mentioned above, such as the TME and dynamic PD-L1 expression, and alternative biomarkers, such as tumor mutational burden (TMB), mismatch repair (MSI), and immune cell infiltration, may all play a role in ICI response, in addition to PD-L1 expression [80,81]. In particular, Richter I. et al. retrospectively studied PD-L1 expression in 33 metastatic castration-resistant PCa patients treated with Enzalutamide. Results showed good tolerability with predominantly G1-2 toxicity, with a median progression-free survival (PFS) of 7.0 months and overall survival (OS) of 8.4 months. Shorter OS was observed in patients with decreasing hemoglobin levels during treatment and those with GS 8-10. The study concluded that PD-L1 expression as a potential predictive biomarker was not confirmed [82].

mCRPC is resistant to immune checkpoint therapy due to few tumor-infiltrating T cells. Ipilimumab and anti-PD-1/PD-L1 monotherapy have not shown significant benefits. A large trial with anti-CTLA-4 plus anti-PD-1 was conducted, with objective response rates of 25% and 10%, and median overall survival of 19.0 and 15.2 months, respectively. Four patients had complete responses, and exploratory studies identified potential biomarkers of response. Grade 3–4 treatment-related adverse events occurred in approximately 42–53% of patients [40].

## 12. PD-L1 as a Prognostic Biomarker

With conflicting findings reported in the literature, the predictive significance of PD-L1 expression in PCa has been highly debated. Several studies have shown that increased PD-L1 expression, whether on tumor or immune cells, is related to a poor prognosis, including shorter disease-free survival, biochemical recurrence-free survival, and overall survival [42,47,83].

However, some investigations have shown no significant relationship between PD-L1 expression and clinical outcomes [84,85].

These discrepancies may be attributed to several factors, such as variations in research design, patient selection criteria, and sample sizes, which may all have an impact on the outcomes. The use of diverse PD-L1 antibodies and scoring methods may introduce heterogeneity into PD-L1 evaluation.

Also, the lack of standardized cut-off values to define PD-L1 positivity can lead to inconsistent results and hinder the interpretation of findings [80].

## 13. Pathological Stage, Lymph Node Metastasis, and Surgical Margins

There is evidence linking PD-L1 expression to features indicative of locally advanced and metastatic disease. In terms of pathological stage (pT), a consistent finding across multiple studies and meta-analyses is that higher PD-L1 expression is associated with a more advanced pathological T stage (i.e., a greater extent of the primary tumor). He J. et al. retrospectively studied 96 cases of PCa and 44 controls with benign hyperplasia, and they found that PD-1/PD-L1 expression was significantly higher in cancer cases compared to benign tissues. PD-L1 expression in tumor cells or lymphocytes was associated with GS, but not with age, preoperative PSA level, clinical T-stage, lymph node metastasis, and risk factor grade. There was no correlation between tumor cells’ and lymphocytes’ high expression of PD-1 and PD-L1. The study concludes that PD-L1 expression is highly prevalent in PCa and may be a co-factor associated with its progression [86].

PD-L1 expression has been assessed in both primary and metastatic PCa, with a few significant variations found between the two scenarios. In primary PCa, tumor cell PD-L1 expression is typically low. However, PD-L1 expression on immune cells, notably TILs, is more common, indicating a continuous immune response in the primary tumor microenvironment. In metastatic PCa, especially mCRPC, PD-L1 expression on both tumor and immune cells is greater than in initial tumors. This might be due to the selection of more aggressive and immunoevasive clones during metastatic development, or the effect of the metastatic microenvironment on PD-L1 expression [87,88]. Shaw et al. [89] reported that PD-L1 positivity was more common in “high-risk” localized PCs (13/50 cases, 26%) and metastatic PCs (7/41 cases, 17%). In some studies, PD-L1 expression was more often seen in PCs with lymph node metastases, sometimes reaching a statistically significant association. For example, Xian et al. found (n = 279; *p* = 0.0294) [56] and Iacovelli et al. found that lymph node metastases were more frequent in PCs expressing PD-L1 in ≥1% of tumor cells (93% vs. 65%; *p* = 0.05; n = 32); this difference became significant when considering a ≥5% cut-off for PD-L1 positivity (60% vs. 40%; *p* = 0.044) [48].

Regarding lymph node metastasis (pN), the relationship between PD-L1 and lymph node involvement is suggestive of a positive correlation. Petitprez F. et al., analyzing 51 PCa patients, found that those with lymph node invasion at radical prostatectomy were at higher risk of tumor recurrence and required immediate androgen deprivation therapy. The study found that patients with PD-L1+ tumor cells had shorter metastasis-free survival and a four-fold risk of distant metastases. PD-L1 expression was significantly associated with CD8+ T-cell density, but not with CD20+ B-cell density. These findings suggest PD-L1 and CD8 may be useful biomarkers for high-risk patients [90].

## 14. Histological Variants and Molecular Subtypes

PCa is not a single disease, and PD-L1 expression varies markedly among its different histological and molecular subtypes. This variation provides important clues about the different biological drivers of PD-L1 expression. Regarding histological variants, the majority of PCa cases are conventional acinar adenocarcinomas, where the overall PD-L1 positivity rate is estimated to be around 29%. However, expression is significantly higher in more aggressive, and often androgen-independent, histological variants. The same reviews report a positivity rate of approximately 46% in neuroendocrine carcinomas/tumors and 42.9% in small cell carcinomas of the prostate. In contrast, the less aggressive ductal adenocarcinoma subtype shows a lower positivity rate of around 7%. This differential expression strongly suggests that the PD-1/PD-L1 pathway may be a particularly critical immune escape mechanism for the most aggressive and difficult-to-treat neuroendocrine variants of PCa [40,91].

In terms of molecular subtypes, PCa comprises a varied set of tumors with distinctive molecular and clinical features. PD-L1 expression has been assessed in different PCa subtypes, showing potential associations with particular molecular changes and treatment vulnerabilities. Prostate malignancies with DNA repair abnormalities, such as BRCA2 mutations or microsatellite instability (MSI), had greater PD-L1 expression than tumors without these mutations. This finding implies that DNA repair errors may cause enhanced neoantigen presentation and a more immunogenic tumor microenvironment, resulting in PD-L1 overexpression as an adaptive immune resistance strategy. For instance, tumors harboring mutations in the SPOP gene, a common event in primary PCa, have been shown to exhibit strong and diffuse PD-L1 staining. This expression is often accompanied by a decrease in TILs, strongly pointing to a constitutive, oncogene-driven mechanism of upregulation rather than an adaptive immune response [36]. In contrast, cases characterized by Mismatch Repair Deficiency (dMMR) or Microsatellite Instability-High (MSI-H), specifically, tumors with defects in the DNA mismatch repair system (dMMR) or the resultant high levels of microsatellite instability (MSI-H), are significantly more likely to be PD-L1 positive. In these tumors, the defective DNA repair machinery leads to the accumulation of thousands of mutations, creating a high TMB and a rich source of neoantigens. This high neoantigen load provokes a strong T-cell response, which in turn drives adaptive, IFN-γ-mediated PD-L1 expression. This subtype represents a classic “hot” tumor phenotype within the generally “cold” landscape of PCa [36,92,93,94,95].

The main adverse histopathological and molecular correlates of PD-L1 expression in PCa are summarized in Table 2.

## 15. The Combination Therapy

The central goal of modern therapeutic development in PCa immunotherapy is to convert immunologically “cold,” non-responsive tumors into “hot” inflamed tumors that are susceptible to checkpoint blockade. This “TME priming” is the cornerstone of the combination strategies currently dominating the clinical trial landscape. Liu YT et al. investigated the drivers of T-cell infiltration that are crucial for ICI efficacy and the strategies to transform “cold tumors” into “hot tumors,” promoting increased T-cell infiltration, such as combination therapy of radiotherapy or chemotherapy with immunotherapy [96].

Androgen deprivation therapy (ADT) and potent androgen receptor (AR) signaling inhibitors like enzalutamide and abiraterone are the backbone of systemic therapy for advanced PCa. Preclinical and clinical evidence show that these agents can induce an inflammatory response in the TME, increase T-cell infiltration, and upregulate PD-L1 expression, providing a strong biological rationale for combining them with ICIs. The KEYNOTE-365 and KEYNOTE-199 trials investigated pembrolizumab in various combinations, including with enzalutamide, showing some durable responses in heavily pretreated mCRPC patients. However, the phase III IMbassador250 trial, which combined atezolizumab with enzalutamide, failed to show an overall survival benefit compared to enzalutamide alone. These mixed results underscore that while the combination is rational, success will likely depend on better patient selection using refined biomarkers [39,58,97].

Key clinical trials of ICIs in mCRPC, together with the variable role assigned to PD-L1 as a biomarker, are summarized in Table 3.

## 16. The Search for a Better Biomarker

The limitations of PD-L1 IHC as a standalone biomarker have catalyzed an urgent and intensive search for more robust, reliable, and clinically actionable biomarkers to guide immunotherapy in PCa. One established target is dMMR/MSI-H. The presence of dMMR/MSI-H is the first and only FDA-approved tissue-agnostic biomarker for pembrolizumab. It identifies a small (3–5%) but critically important subset of PCa patients whose tumors have an extremely high TMB and are highly responsive to ICI monotherapy; therefore, all patients with metastatic PCa should be tested for this biomarker [98,99]. Another relevant factor is tumor mutational burden (TMB). High TMB, a quantitative measure of the number of somatic mutations per megabase of DNA, is another marker of high neoantigen load. While not yet a standard-of-care biomarker in PCa, it is being actively investigated, often as a complementary marker to PD-L1, to identify patients who may benefit from ICIs [100]. Moreover, researchers are focusing on genomic signatures, where the presence of specific genomic alterations, such as mutations in DDR genes (BRCA1/2, ATM) or SPOP, may define distinct biological subtypes of PCa with differential sensitivity to ICI-based combinations [36,101]. Finally, moving beyond single analytes, researchers are developing multi-gene-expression profiles. Signatures that reflect the activity of the IFN-γ signaling pathway or quantify the degree of T-cell inflammation within the TME may provide a more holistic and accurate assessment of the tumor’s immune status than PD-L1 IHC alone [102].

## 17. Novel Frontiers: Liquid Biopsies and New Targets

Particularly promising are two new fields that could completely revolutionize the use of immunotherapy for PCa: the use of liquid biopsies and the neuro-immune axis.

Regarding liquid biopsies, the limitations of tissue biopsies—being invasive and unable to capture spatial and temporal heterogeneity—can potentially be overcome by this approach. Clinicians can get a real-time, non-invasive picture of the tumor’s molecular state by examining circulating tumor cells (CTCs), circulating tumor DNA (ctDNA), or tumor-derived exosomes from a straightforward blood sample. Research has previously shown that PD-L1 can be found on CTCs and in exosomes, and that therapy can alter its levels. A genuinely individualized strategy may be made possible by the capacity to dynamically monitor PD-L1 expression and other biomarkers (such as TMB from ctDNA) during a patient’s course of treatment. This would allow therapy to be modified in response to the tumor’s changing biology [103].

With respect to the PD-1/PD-L1 axis, liquid biopsy readouts may include PD-L1 expression on circulating tumor cells, PD-L1 carried by extracellular vesicles/exosomes, and ctDNA-derived genomic context (e.g., MSI/dMMR, TMB, CDK12 alterations), which can be integrated with immune phenotyping. These modalities are particularly attractive for longitudinal sampling, allowing PD-L1 dynamics to be tracked during ADT, ARSI, radiotherapy, and ICI-based combinations, and may reduce the risk of misclassification from a single archival tissue block [28,103].

In parallel, targeting the neuro-immune axis represents a whole new aspect of immunosuppression in PCa that has been revealed by the groundbreaking finding of elevated PD-L1 expression on tumor-associated nerves (TANs). According to this research, the nervous system actively works with the tumor to protect it from immune attack rather than acting as a passive spectator. Disrupting this abnormal neuro-immune interaction brings up a new therapeutic avenue. In order to break down this special immune-privileged niche and expose the tumor to immune destruction, future therapies may combine ICIs with medications that block the physical connection between tumor cells and nerves or target nerve signaling pathways [26].

These future directions are coming together to suggest a new paradigm for PCa immunotherapy. It will probably be a multi-step, customized approach that goes beyond a “one-size-fits-all” methodology. In order to determine the best therapeutic window for intervention, this may entail initial TME “priming” with conventional therapies such as ADT or radiation; dynamic immune response monitoring with liquid biopsies; and the administration of carefully chosen combination immunotherapies based on the patient’s unique tumor biology (e.g., dMMR status, DDR deficiency, or evidence of neuro-immune suppression). The function of PD-L1 will change under this new paradigm from that of a static, imperfect gatekeeper to that of a dynamic indicator of therapeutic opportunity that directs the wise sequencing and pairing of therapies.

An overview of emerging biomarkers and strategies beyond PD-L1 in PCa is provided in Table 4.

## 18. Future Perspectives and Conclusions

PD-L1 in PCa is a topic of great clinical significance and complexity. A clear, if nuanced, picture emerges from this review of the extant evidence. PD-L1 expression is linked to features of biologically aggressive disease, indicating that it is not a random event. Higher PD-L1 expression is associated with adverse clinicopathological characteristics, including aggressive histological variants (e.g., neuroendocrine carcinoma), lymph node involvement, advanced pathological stage, higher Gleason grade groups, and positive surgical margins. In addition, the identification of elevated PD-L1 expression on tumor-associated neurons has revealed a potential new pathway of neuro-immune suppression in PCa progression.

However, there are two major obstacles that seriously impair the ability of these findings to become a clinically useful, stand-alone biomarker. First, there is a methodological dilemma. Different antibody clones, scoring algorithms, positivity cut-offs, and specimen handling techniques are all examples of an extreme lack of consistency in PD-L1 detection, which has led to a disjointed and conflicting body of evidence. This metrological turmoil has hindered the development of a validated, repeatable assay for clinical application in PCa and has made it practically impossible to compare results across investigations.

The underlying biology of the illness presents the second difficulty. PD-L1 expression is decoupled from the presence of a strong, pre-existing anti-tumor immune response by the largely “cold” or non-inflammatory TME of the majority of prostate tumors. This explains why the independent predictive usefulness of PD-L1 status is still quite controversial and why it has been shown to be a poor predictor of response to ICI monotherapy.

Therefore, the path forward for realizing the potential of the PD-1/PD-L1 axis in PCa requires a significant strategic shift. This multi-pronged approach must include standardization; a concerted, field-wide effort, akin to the “Blueprint Project” in lung cancer, is urgently needed to harmonize and validate PD-L1 testing methodologies for PCa, representing a prerequisite for any meaningful clinical development. Furthermore, contextualization is essential, as PD-L1 expression should not be interpreted in a vacuum. Its clinical utility will likely be realized only when it is integrated into a multi-modal biomarker panel that includes established genomic markers (dMMR/MSI-H, TMB, DDR gene status) and comprehensive characterization of the TME. Moreover, the therapeutic future for the vast majority of PCa patients lies not in ICI monotherapy but in rationally designed combination strategies. Therapies that can effectively “heat up” the cold TME (such as androgen deprivation therapy, radiotherapy, and PARP inhibitors) are essential partners for checkpoint inhibitors. Finally, the concept of dynamism must be embraced: the assessment of PD-L1 must evolve from a static, one-time measurement on a pre-treatment tissue biopsy to a dynamic, longitudinal process. The adoption of liquid biopsies to monitor the evolution of PD-L1 and other biomarkers in real time will be critical for guiding the timing, sequencing, and selection of these complex combination therapies.

As a pragmatic pathway toward standardization, future PCa studies and clinical trials should preferentially use clinically validated assays on their intended platforms (e.g., 22C3/28-8 or SP263), report tumor-cell and immune-cell compartments with explicit definitions, and provide both continuous values and pre-specified categorical cut-offs for TPS and/or CPS (Table 1). Equally important is transparent reporting of pre-analytical variables (sampling site, fixation, decalcification, and block age), together with independent quality assurance and inter-laboratory concordance exercises. Where tissue is limited or spatial context is essential, multiplex immunofluorescence and digital pathology approaches may offer a feasible route to harmonized, context-aware immune profiling.

A further step is to learn explicitly from tumor types in which PD-L1-guided immunotherapy has been developed most extensively. In NSCLC, meta-analyses have shown that higher PD-L1 expression is associated with greater benefit from PD-1/PD-L1 blockade (including in chemo-immunotherapy combinations), while also highlighting the limitations of PD-L1 as a stand-alone binary gatekeeper [104,105]. Melanoma similarly illustrates that clinically meaningful responses can occur even in PD-L1-negative disease, consistent with its generally more inflamed biology and reinforcing the need to interpret PD-L1 within integrated immune and genomic profiles rather than in isolation [17]. These experiences support a shift in PCa from single-marker thinking to standardized assays combined with composite biomarkers (TMB/MSI, DNA damage response (DDR) status, immune gene signatures, and spatial TME phenotyping) that can better guide trial design and patient management.

In conclusion, PD-L1 in PCa should not be viewed as a simple lock-and-key biomarker. It is a complex and dynamic indicator of tumor biology, immune interaction, and therapeutic adaptation. While its current utility is limited by the significant hurdles of measurement and biological context, a deeper and more nuanced understanding of its role is essential for unlocking the full potential of immunotherapy and improving outcomes for patients with this challenging disease.

## Figures and Tables

**Table 1:** Comparison of PD-L1 immunohistochemistry (IHC) clones and scoring systems in PCa studies.

Clone (Vendor)	Typical Platform	Epitope Tendency	Common Scoring in the Literature
22C3 (Dako/Agilent)	Dako/Agilent	Extracellular	TPS and/or CPS
28-8 (Dako/Agilent)	Dako/Agilent	Extracellular	TPS and/or CPS
SP142 (Ventana/Roche)	Ventana/Roche	Cytoplasmic	IC score and/or CPS
SP263 (Ventana/Roche)	Ventana/Roche	Cytoplasmic	TPS and/or CPS
E1L3N/5H1/ABM4E54 (Research Use Only, RUO)	Varies	Varies	Variable/qualitative

**Table 2:** Correlation of PD-L1 expression with adverse histopathological features in PCa.

Advanced Pathological Stage (pT3/4)
Lymph Node Metastasis (pN+)
Aggressive Histotypes (Neuroendocrine/Small Cell)
Molecular Subtypes (dMMR/MSI-H)
Molecular Subtypes (*SPOP*-mutant)

**Table 3:** Summary of Key Clinical Trials with Immune Checkpoint Inhibitors (ICIs) in mCRPC.

Trial Name	Agent(s) and Treatment Line	Population and Cohorts	Key Results (ORR/OS)	PD-L1 Biomarker Role
KEYNOTE-028 (Phase Ib)	Pembrolizumab (mono)	Advanced solid tumors PD-L1+ (including PCa).	ORR: ~17% (in PCa cohort).	Inclusion criterion (only PD-L1+).
KEYNOTE-199 (Phase II)	Pembrolizumab (mono) Post-docetaxel and hormonal therapy.	Cohort 1: PD-L1+ (CPS ≥1) Cohort 2: PD-L1 neg Cohort 3: Bone metastases (indep. of PD-L1)	ORR Cohort 1: 5% ORR Cohort 2: 3% Median OS longer in Cohort 1 vs. 2 (9.5 vs. 7.9 months).	Trend towards better response and OS in PD-L1+ patients (CPS ≥1), but activity is modest and not exclusive to positives.
CheckMate 650 (Phase II)	Nivolumab + Ipilimumab (Combo) mCRPC.	Cohort 1: Pre-chemotherapy Cohort 2: Post-chemotherapy	ORR Cohort 1: 25% ORR Cohort 2: 10% High toxicity (G3-4: 40–50%).	Trend towards better responses in patients with PD-L1 expression (≥1%), but responses also observed in negatives. Predictive value not definitive.
IMbassador250 (Phase III)	Atezolizumab + Enzalutamide vs. Enzalutamide. Post-abiraterone (pre-chemo).	mCRPC not selected for PD-L1.	Primary endpoint (OS) not met. No significant benefit from adding Atezolizumab.	Subgroup analysis by PD-L1 status (IC < 1%, 1–4%, ≥5%) showed no differential benefit in OS or PFS.
KEYNOTE-365/PROpel (Phase Ib/II/III)	Pembrolizumab + Olaparib (PARPi)	mCRPC (various settings, including unselected for HRR).	Data suggest potential rPFS benefit from the combination compared to Olaparib alone or hormonal therapy.	The role of PD-L1 as a selection biomarker for these combinations is still being defined; efficacy seems driven by biological synergy.

**Table 4:** Emerging biomarkers and strategies beyond PD-L1 in PCa.

Biomarker/Strategy	Biological Rationale in PCa	Typical Detection Method	Current Clinical Status (FDA/Experimental)
dMMR/MSI-H (Mismatch Repair Deficiency/High Microsatellite Instability)	High rate of somatic mutations leading to the generation of many neoantigen, making the tumor “Hot” and sensitive to immunotherapy.	Immunohistochemistry (for MMR proteins) or NGS Sequencing (for MSI).	FDA Approved (tumor-agnostic) for pembrolizumab in patients with unresectable or metastatic dMMR/MSI-H solid tumors experiencing progression.
TMB-High (High Tumor Mutational Burden)	Higher probability of presenting immunogenic neoantigens that can be recognized by T cells.	NGS Sequencing (e.g., FoundationOne CDx).	FDA Approved (tumor-agnostic) for pembrolizumab in solid tumors with TMB ≥10 mut/Mb.
DDR Mutations (e.g., *BRCA1/2, ATM*)	DNA repair deficiency (HRR) causes genomic instability and a potential “cyclosporine-like” signature. Strong rationale for synergy between PARP inhibitors and ICIs (e.g., accumulation of DNA damage stimulating the STING pathway).	NGS Sequencing (gene panels on tissue or blood).	PARPi + ICI combinations in advanced clinical trial stages (e.g., PROpel trial).
*SPOP* Mutations	The mutated form of SPOP fails to ubiquitinate PD-L1 for degradation, leading to high constitutive expression of PD-L1 and potential immune evasion.	NGS Sequencing.	Experimental. Identifies a specific molecular subgroup with high PD-L1 expression.
Liquid Biopsy (CTCs and Exosomes)	Allows for dynamic and non-invasive monitoring of PD-L1 expression, overcoming limitations of tumor heterogeneity and the difficulty of re-biopsying bone metastases.	Isolation from peripheral blood and analysis (IHC, PCR, cytometry).	Experimental/Clinical validation ongoing.
Neuro-Immune Axis	Nerve fibers infiltrating the tumor can express PD-L1, contributing to an immunosuppressive “sanctuary” that protects tumor cells (nerve-mediated immune evasion).	Complex Immunohistochemistry (neural markers + PD-L1).	Preclinical/Recent discovery. New potential therapeutic pathway.

Abbreviations: ADT, androgen deprivation therapy; AR, androgen receptor; CTCs, circulating tumor cells; ctDNA, circulating tumor DNA; CXCL9/10/11, C-X-C motif chemokine ligand 9/10/11; dMMR, mismatch repair deficiency; DDR, DNA damage response; DTX, docetaxel; EVs, extracellular vesicles; IFN-γ, interferon gamma; ICI, immune checkpoint inhibitor; IHC, immunohistochemistry; IL, interleukin; IRF1, interferon regulatory factor 1; mCRPC, metastatic castration-resistant prostate cancer; mHSPC, metastatic hormone-sensitive prostate cancer; MSI, microsatellite instability; MSI-H, microsatellite instability–high; MDSCs, myeloid-derived suppressor cells; NK, natural killer (cells); NSCLC, non-small cell lung cancer; OS, overall survival; PARPi, poly(ADP-ribose) polymerase inhibitor; PCa, prostate cancer; PD-1, programmed cell death protein 1; PD-L1, programmed death-ligand 1; PFS, progression-free survival; PSA, prostate-specific antigen; RT, radiotherapy; TILs, tumor-infiltrating lymphocytes; TMB, tumor mutational burden; TME, tumor microenvironment; TNBC, triple-negative breast cancer; Treg, regulatory T (cells); VEGF, vascular endothelial growth factor.

## Data Availability

No new data were created or analyzed in this study. Data sharing is not applicable to this article.
